# Conditioned Media of Adipose-Derived Stem Cells Suppresses Sidestream Cigarette Smoke Extract Induced Cell Death and Epithelial-Mesenchymal Transition in Lung Epithelial Cells

**DOI:** 10.3390/ijms222112069

**Published:** 2021-11-08

**Authors:** Tzu-Yin Chen, Chia-Hao Liu, Tsung-Hsien Chen, Mei-Ru Chen, Shan-Wen Liu, Pinpin Lin, Kurt Ming-Chao Lin

**Affiliations:** 1 Institute of Biomedical Engineering and Nanomedicine, National Health Research Institutes, Zhunan 35053, Taiwan; chenty@nhri.edu.tw (T.-Y.C.); chliu1280@gmail.com (C.-H.L.); cych13794@gmail.com (T.-H.C.); 940501@nhri.edu.tw (M.-R.C.); jwliu@nhri.edu.tw (S.-W.L.); 2 Ditmanson Medical Foundation Chia-Yi Christian Hospital, Chia-Yi 600566, Taiwan; 3 Institute of Population Health, National Health Research Institutes, Zhunan 35053, Taiwan; 4 National Institute of Environmental Health Sciences, National Health Research Institutes, Zhunan 35053, Taiwan; pplin@nhri.edu.tw

**Keywords:** adipose-derived stem cell, conditioned medium, epithelial–mesenchymal transition (EMT), cigarette smoke extract, COPD, TGF-β1

## Abstract

The role of the epithelial–mesenchymal transition (EMT) in lung epithelial cells is increasingly being recognized as a key stage in the development of COPD, fibrosis, and lung cancers, which are all highly associated with cigarette smoking and with exposure to second-hand smoke. Using the exposure of human lung cancer epithelial A549 cells and non-cancerous Beas-2B cells to sidestream cigarette smoke extract (CSE) as a model, we studied the protective effects of adipose-derived stem cell-conditioned medium (ADSC-CM) against CSE-induced cell death and EMT. CSE dose-dependently induced cell death, decreased epithelial markers, and increased the expression of mesenchymal markers. Upstream regulator analysis of differentially expressed genes after CSE exposure revealed similar pathways as those observed in typical EMT induced by TGF-β1. CSE-induced cell death was clearly attenuated by ADSC-CM but not by other control media, such as a pass-through fraction of ADSC-CM or A549-CM. ADSC-CM effectively inhibited CSE-induced EMT and was able to reverse the gradual loss of epithelial marker expression associated with TGF-β1 treatment. CSE or TGF-β1 enhanced the speed of A549 migration by 2- to 3-fold, and ADSC-CM was effective in blocking the cell migration induced by either agent. Future work will build on the results of this in vitro study by defining the molecular mechanisms through which ADSC-CM protects lung epithelial cells from EMT induced by toxicants in second-hand smoke.

## 1. Introduction

Cigarette smoking is a major risk factor for many lung diseases, including chronic obstructive pulmonary disease (COPD), asthma, fibrotic diseases, and lung cancers [1,2,3]. Cigarette smoke extract (CSE), with more than 4000 chemical constituents, is known to cause cell injury, apoptosis, inflammation, and cancer [4,5,6,7,8,9,10,11]. The epithelial cells lining the airway that form tight barriers with specialized functions, such as mucus secretion and ciliary clearance, are the first layer of defense against inhaled toxicants. The epithelial-to-mesenchymal transition (EMT) describes a cell transformation process in which epithelial cells transition to more migratory and invasive mesenchymal-type cells [12,13]. EMT has been identified as a key pathological transformation of lung epithelial cells that contributes to the development of COPD, lung fibrosis, and lung cancer [13,14,15,16,17]. Studies have demonstrated that mesenchymal markers, such as vimentin, α-SMA, and *S100A4*, were increased in the airways of COPD patients and active smokers [18,19]. Compared to the well-illustrated roles of EMT in carcinogenesis, the molecular pathways leading to elevated EMT in COPD patients [20,21,22] or by exposure to CSE in vitro [23,24,25] have not been fully elucidated. Among the pathways that have been identified to be involved in EMT, the activation of the TGF-β family and the Wnt/β-catenin signaling pathways have been implicated in EMT associated with COPD or with exposure to CSE collected from mainstream cigarette smoke [16,18,23,26,27].

A significant and increasing percentage of COPD or lung cancer patients are non-smokers, but these patients are exposed to second-hand smoke from environment [28,29]. There are evidence that second-hand cigarette smoke collected from sidestream smoke contains more toxic substances than mainstream smoke [30]. Unlike mainstream smoke, the in vitro or in vivo cellular response to secondhand smoke exposure has been studied in frequently [31], and whether sidestream CSE induces EMT in pulmonary epithelial cells has not been reported before. 

Adipose-derived stem cells (ADSCs) are the most abundant type of stem cell in adults, and there is ongoing research focused on the therapeutic applications of ADSCs. Similar to mesenchymal stem cells (MSC) derived from bone marrow (BMMSC), ADSCs display multilineage potential and an immune-regulatory capacity. The therapeutic potential of ADSCs in the treatment of various diseases has been demonstrated using various experimental models. The transplantation of ADSCs via intravenous injection in mice reduced the infiltration of inflammatory cells, lung cell death, and airway enlargement in a cigarette smoke exposure-induced emphysema model [32,33,34]. Multiple clinical trials involving MSCs have demonstrated the safety of MSC implantation, but the clinical benefits are not yet conclusive, e.g., the intravenous infusion of adult MSCs as a treatment for COPD has not demonstrated clinical efficacy [35], indicating that more research is needed to explore the full therapeutic potential of ADSCs [36]. The therapeutic effects of a conditioned medium cultured with ADSCs (ADSC-CM) or MSCs from other sources were widely explored with outcomes that included angiogenesis and lung tissue repair [34,36]. Various growth factors, such as transforming growth factor β (TGF-β), fibroblast growth factor (FGF), keratinocyte growth factor (KGF), hepatocyte growth factor (HGF), vascular endothelial growth factor (VEGF), and stem cell factor (SCF), have been identified in ADSC-CM [37,38,39]. In cell culture and animal models, the benefits of ADSC-CMs were partially mediated by the growth factors that were present in the conditioned medium [37,38,39,40]. The potentials of ADSCs or their conditioned mediums in cancer therapy remain controversial, as both anti-tumor and pro-tumor effects have been reported after implanting MSCs or in coculture studies with tumor cells [36]. The anti-tumor effect by CM has been linked to the inhibition of the tumor cell cycle, while tumor-trophic effects including, inducing EMT in cancer cells, have been reported to involve a direct MSC–tumor interaction that stimulates the inflammatory cytokines and metalloproteinases that released are by MSCs and that promote tumor migration [36,41].

In this study, we investigated whether CSE collected from sidestream cigarette smoke induced lung epithelial cell injury and explored the upstream signaling pathways underlying cell injury and EMT induction. By comparing the results with typical EMT induced by TGF-β1, we identified signaling pathways that are uniquely induced by CSE and pathways that are also involved in TGF-β1 stimulation. We then explored the ADSC-CM-mediated protection of epithelial cells through the inhibition of EMT.

## 2. Results

### 2.1. CSE Induces Lung Epithelial Cell Death in Concentration- and Serum-Dependent Manners

**Figure 1:**
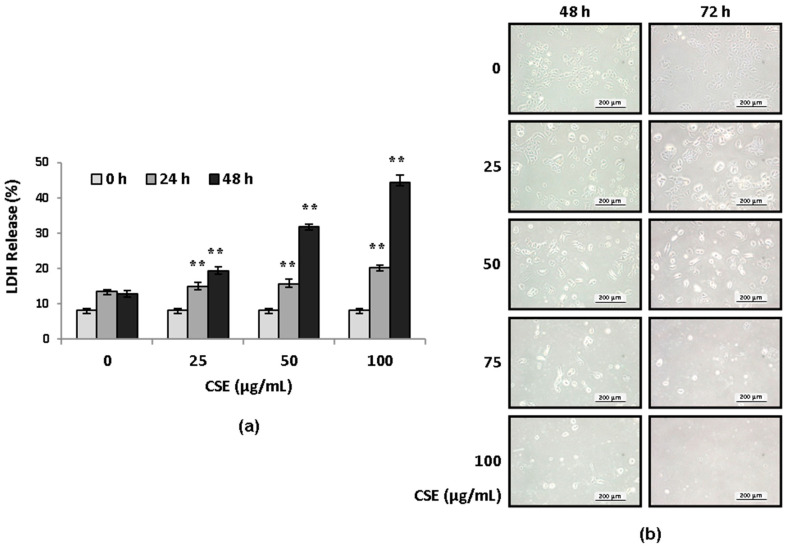
Cytotoxicity induced by CSE in A549 cells. (**a**) A549 cells were treated with 25–100 μg/mL CSE for 24 or 48 h, and cytotoxicity was measured by LDH release from cells. The readings were normalized to the maximum LDH activity in cells and medium. ** *p* < 0.01, compared to untreated cells. *N* = 4. (**b**) Images of A549 cells showing cell death after exposure to CSE for 48 or 72 h. CSE, cigarette smoke extract; LDH, lactose dehydrogenase.

Sidestream cigarette smoke extracts were prepared as described in the Section 4. We exposed the human lung epithelial cell line A549 to CSE of various concentrations (from 25 to 100 μg/mL) in standard culture medium containing 10% serum or in serum-free medium and studied the surviving cells by means of the WST-1 assay and indicators of cell death with the LDH release assay at 24, 48, and 72 h. Results of the WST-1 assay showed a reduction in cell viability after CSE exposure in serum- and CSE concentration-dependent manners (data not shown). In the serum-free medium, a CSE concentration of 50 μg/mL or higher significantly reduced A549 cell viability after 24 h, while CSE of 25 μg/mL or less only had a slight effect on cell viability, even after 72 h. In the medium containing 10% serum, the effect of CSE on cell viability was blunted, and high-dose CSE (100 μg/mL) resulted in a significant loss of cell numbers at 72 h after exposure. WST-1 conversion to formazan is an indicator of cell proliferation; therefore, the assay results that indicate less conversion may result from a loss of cell viability, reduced cell proliferation, or both. As increased LDH release from dying cells is a more direct indicator of cytotoxicity, we measured LDH release after CSE exposure. Exposure to a 25 μg/mL or higher concentration of CSE caused a significant increase in LDH release by A549 cells at 24 and 48 h (Figure 1a). Images of A549 cells after exposure to CSE for 48 and 72 h are shown in Figure 1b, demonstrating that CSE of 75 μg/mL or higher resulted in severe cell injury that prevented subsequent biochemical assays, while exposure to CSE of 25 μg/mL or less did not cause significant damage. Thus, in subsequent studies on the protective effects of ADSC-CM, we induced cell injury and EMT via exposure to 50 μg/mL CSE in serum-free medium.

### 2.2. ADSC-Conditioned Medium Protected Cells from CSE-Induced Epithelial Cell Death

**Figure 2:**
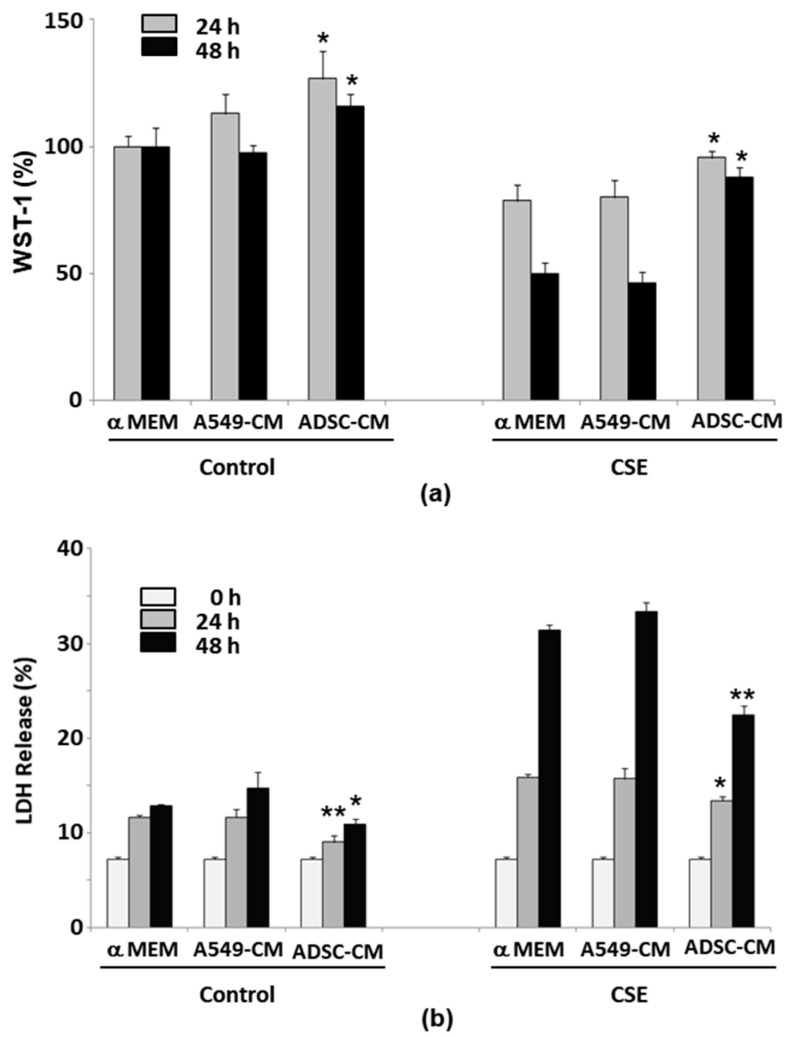
ADSC-CM protects A549 cells from CSE-induced cytotoxicity. (**a**) A549 cells were incubated with standard growth medium (α-MEM), A549-CM, or ADSC-CM for 24 or 48 h (left panel) or under the same culture conditions plus 50 μg/mL CSE (right panel). Cell viability was determined at 24 and 48 h by WST-1 assay and was normalized to that of the α-MEM group. In (**b**), as in (**a**), cytotoxicity was determined by LDH release, as in Figure 1. * *p* < 0.05; ** *p* < 0.01 compared to α-MEM and A549-CM treatment groups at 24 or 48 h. *N* = 4. ADSC, adipose-derived stem cell; CM, conditioned medium; CSE, cigarette smoke extract; LDH, lactose dehydrogenase.

We used concentrated ADSC-CM to explore its ability to protect A549 cells against CSE-induced toxicity and death. The experimental controls for ADSC-CM included the Pass-Through (PT) fraction from the conditioned media concentration step, A549-CM, and pure α-MEM. Each type of medium was concentrated using the same method as was used to prepare the ADSC-CM. We treated the A549 cells by culturing them in various media, both with or without 50 μg/mL CSE, and assessed cell viability and cytotoxicity using the WST-1 assay and the LDH assay, respectively. The viability of the cells cultured with A549-CM showed a slight but non-significant increase at 24 h, while it increased significantly with ADSC-CM compared to cells cultured in α-MEM (Figure 2a) or ADSC-PT medium (data not shown). At 48 h, the cells cultured in A549-CM no longer showed more increased viability than the cells grown in α-MEM, whereas higher cell viability was found in the cells cultured in ADSC-CM than in cells that had been cultured in other types of media (Figure 2a). CSE treatment significantly reduced the cell survival at 48 h to approximately half the level observed for cells cultured in α-MEM or A549-CM without CSE exposure. In contrast, A549 cells cultured in ADSC-CM and that had been exposed to CSE maintained cell viability of at least 80% of the level observed for cells without CSE (Figure 2a). Culturing the cells in A549-CM was not significantly different versus serum-free α-MEM after exposure to CSE in both the WST-1 and LDH release assays (Figure 2a,b). The use of ADSC-CM significantly reduced the amount of LDH that was released the by cells after they had been cultured in serum-free medium as well as after CSE treatment (Figure 2b). These results demonstrate that ADSC-CM, but not its PT-fraction or A549-CM, provides substantial protection against CSE-induced toxicity in A549 cells.

### 2.3. ADSC-CM Reduces CSE-Induced EMT in A549 Cells

**Figure 3:**
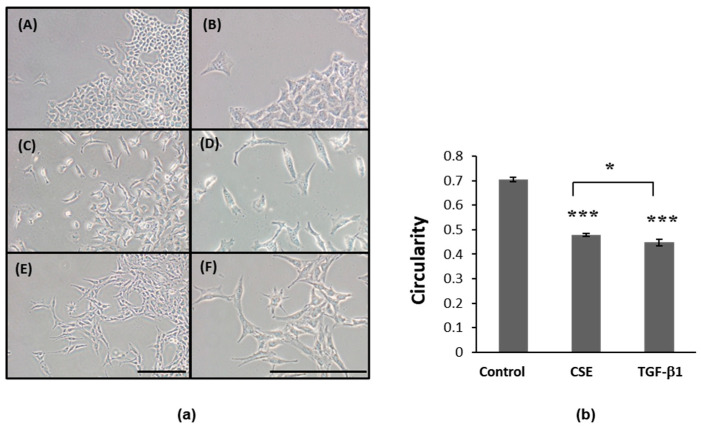
Morphological changes in A549 cells responding to CSE or TGF-β1. (**a**) A549 cells changed from tightly packed cobblestone epithelial-like shapes (A, B) to more isolated, irregular, and elongated shapes after 72 h of exposure to CSE (C, D) or TGF-β1 (E, F). (**b**) Computed circularity of cells in (**a**). A total of 100 cells from each culture condition were measured, and the results comprise data from three independent experiments. Scale bar = 200 μm, * *p* < 0.05, comparing CSE and TGF-β1; *** *p* < 6.0 × 10^−5^ compared to control. CSE, cigarette smoke extract.

After the A549 cells were exposed to CSE, we observed any morphological changes that occurred, including those to the shape and density transition from tightly packed polygonal cells to elongated and irregularly shaped cells that had less contact with neighboring cells (Figure 3a). These morphological changes are characteristic features of EMT. We measured the changes in the cellular morphology using the circularity index, scoring the cells between 0 (linear shape) and 1 (perfect round shape), as well as compared them with the cells that had been treated with 5 ng/mL TGF-β1 to induce EMT as a positive control. Treatment with either CSE or TGF-β1 decreased the circularity of the A549 cells, inducing the transition to more elongated and irregularly shaped cells, with circularity index scores reduced from 0.704 (control cells) to 0.479 or 0.447 after CSE or TGF-β1 treatment, respectively (Figure 3b). Thus, we hypothesized that CSE induces EMT in A549 cells and investigated whether ADSC-CM, which reduces CSE-induced cell death, also regulates CSE-induced EMT.

**Figure 4:**
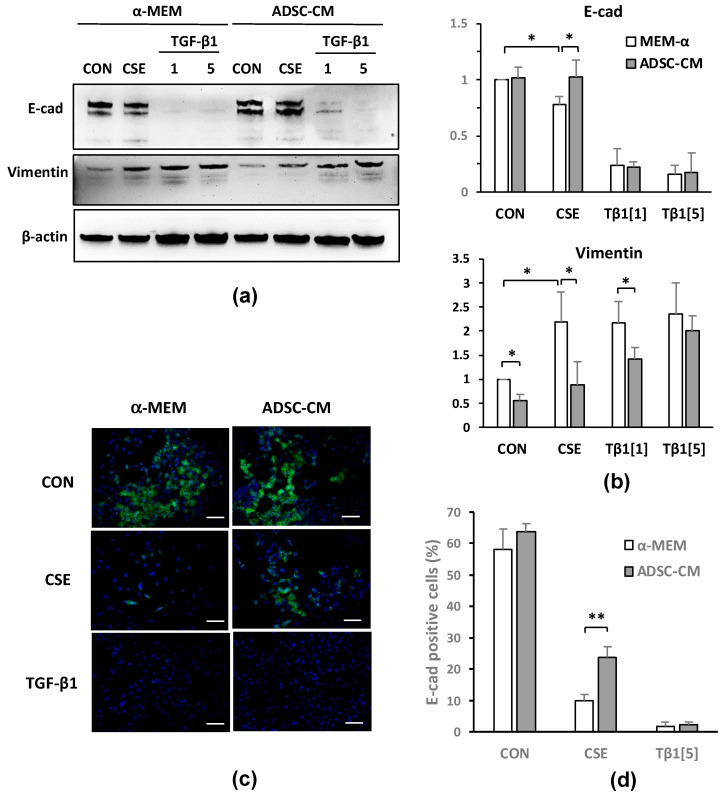
CSE- and TGF-β1-induced EMT in A549 cells blocked by ADSC-CM. (**a**) E-cadherin (E-cad), vimentin, and β-actin expression by immunoblotting in A549 cells treated with 0 or 50 μg/mL CSE and 1 or 5 ng/mL TGF-β1 (Tβ1[1], Tβ1[5]) for 72 h. Both E-cad and vimentin appeared as multiple bands in Western blot. Increased vimentin and decreased E-cad expression are markers of EMT. (**b**) Summary of densitometry analysis of E-cad and vimentin expression levels normalized to the level of β-actin. The results shown were summarized from five independent experiments showing values normalized to α-MEM control sample. * *p* < 0.05. (**c**) Immunofluorescent staining of E-cadherin in A549 cells cultured in α-MEM or ADSC-CM and treated with CSE or 5 ng/mL TGF-β1. Scale bar = 100 μm. (**d**) Percentage of cells in (**c**) that showed positive staining for E-cadherin. Images from three independent experiments were analyzed by ImageJ. ** *p* < 0.01 compared to a-MEM+CSE group. ADSC, adipose-derived stem cell; CM, conditioned medium; CON, control; CSE, cigarette smoke extract.

We treated A549 cells with α-MEM containing 1% FBS plus 0, 25, or 50 μg/mL CSE or 5 ng/mL TGF-β1 for 72 h, followed by immunoblotting and immunofluorescent staining for epithelial and mesenchymal markers. The expression of the epithelial cell marker E-cadherin (E-cad) was decreased, and the expression of mesenchymal markers such as vimentin and α-SMA was increased by CSE treatment in a dose-dependent manner (data not shown). Next, we explored whether ADSC-CM could regulate EMT induction by CSE. We treated cells with 50 μg/mL CSE and 1 or 5 ng/mL of TGF-β1 in either α-MEM or ADSC-CM for 72 h. E-cad expression was found to be decreased, and vimentin expression increased in A549 cells, which was caused by either CSE or TGF-β1, indicating that CSE or TGF-β1 induces EMT in A549 cells. This EMT induction was attenuated by ADSC-CM. CSE-induced or changes in E-cad or vimentin were found to be sensitive to ADSC-CM, which also attenuated the vimentin induction by 1 ng/mL TGF-β1, but not the vimentin induced by 5 ng/mL TGF-β1 (Figure 4a,b). The immunostaining results demonstrated that the E-cad expression in the A549 cells was greatly decreased by either CSE or TGF-β1 treatment and that ADSC-CM allowed the greater retention of cellular E-cad expression after CSE exposure compared to the cells that had been cultured in α-MEM (Figure 4c). The image quantification results summarized in Figure 4d demonstrate that while the percentage of E-cad-positive cells was reduced from 60% in the controls to 10% after CSE exposure, the cells that had been cultured in ADSC-CM maintained an E-cad-positive cell percentage of over 20%. In contrast, ADSC-CM was less effective in preserving E-cad expression after the 5 ng/mL TGF-β1 treatment, as less than 3% of the A549 cells expressed E-cad after 72 h. Although only differing slightly, both the immunoblotting and the immunostaining results support the fact that ADSC-CM inhibits EMT induction in A549 cells due to CSE treatment.

**Figure 5:**
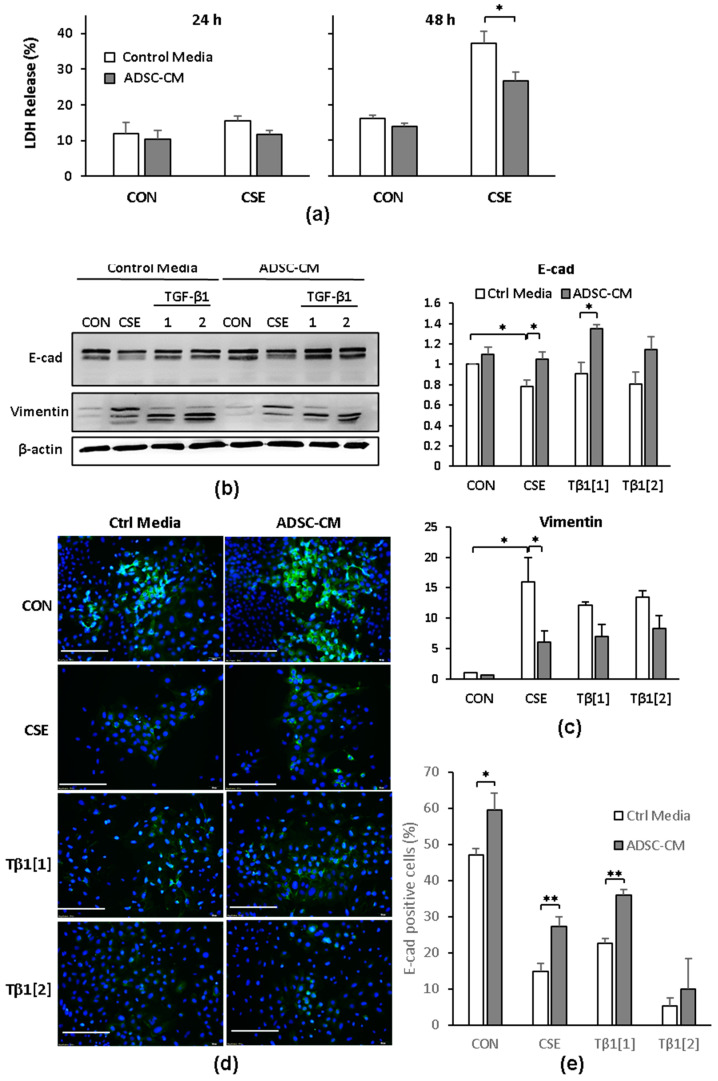
CSE- and TGF-β1-induced cell death and EMT in Beas-2B (B2B) cells blocked by ADSC-CM. (**a**) LDH release by B2B cells in control medium or ADSC-CM kept as control or treated with 50 μg/mL CSE. * *p* < 0.05. (**b**) E-cad, vimentin, and β-actin expression by immunoblotting in B2B cells treated with 0, 50 μg/mL CSE and 1 or 2 ng/mL TGF-β1 (Tβ1[1], Tβ1[2]) for 72 h. (**c**) Summary of densitometry analysis of E-cad and vimentin expression normalized to the level of β-actin. The results shown were summarized from five independent experiments showing values normalized to control medium groups. * *p* < 0.05. (**d**) Immunofluorescent staining of E-cadherin in B2B cells kept in control media or ADSC-CM and treated with CSE and 1 or 2 ng/mL TGF-β1 for 72 h. Scale bar = 200 μm. (**e**) Percentage of cells in (**d**) that showed positive staining for E-cadherin. Images from three independent experiments were analyzed. ** *p* < 0.01 compared to control media group. ADSC, adipose-derived stem cell; CM, conditioned medium; CON, control; CSE, cigarette smoke extract.

A549 cells are lung epithelial cells that originate from the tumorous tissues in which properties relating to EMT may not be the same as they are in non-cancerous cells. Therefore, we investigated the toxicity and induction of EMT by CSE in human non-tumor bronchus/lung epithelial cells and Beas-2B (B2B) cells. We treated the B2B cells as described in Section 4 with 50 μg/mL CSE in the control medium and in medium containing ADSC-CM, and we measured LDH release from the cells after 24 h or 48 h. CSE resulted in strong increases in LDH release after 48 h, which was reduced with the ADSC-CM-containing medium (Figure 5a). In the pilot study, we found that a TGF-β1 dose that was lower than 5 ng/mL was sufficient to induce EMT in B2B cells (data not shown). We treated B2B cells with 50 μg/mL CSE and 1 or 2 ng/mL TGF-β1 in the control media or in medium containing ADSC-CM followed by an assessment of epithelial and mesenchymal marker expression. CSE resulted in the reduction of E-cad in the control medium; that reduction was blunted by ADSC-CM (Figure 5b). Although TGF-β1 did not appear to cause an appreciable E-cad reduction in the Western blots, a rapid disappearance of E-cad expressing cells due to TGF-β1 was clearly observed in the immunostaining results. Compared to culturing in control medium, media containing ADSC-CM resulted in a higher E-cad expression in B2B cells. CSE or TGF-β1 caused a substantial increase of vimentin expression that was significantly reduced by ADSC-CM (Figure 5b,c). The immunostaining of E-cad in B2B cells demonstrated that E-cad expression was greatly decreased by either CSE or TGF-β1 treatment. ADSC-CM allowed the greater retention of cellular E-cad expression after CSE or TGF-β1 exposure compared to B2B cells cultured in control medium (Figure 5d). Image quantification of the immunostaining results is summarized in Figure 5e and shows that the percentage of E-cad-positive cells was reduced by CSE or TGF-β1 and was significantly increased by ADSC-CM. For B2B cells treated with 2 ng/mL TGF-β1, ADSC-CM was less effective in preserving E-cad positive cells. These data suggest that CSE causes cell death and induces EMT in non-cancerous lung epithelial cells, as is also the vase in A549 cells, and CSE-induced cell death and EMT can be suppressed by ADSC-CM.

### 2.4. Gene Expression Profiles of A549 Cells Responding to CSE or TGF-β1

To identify the changes in the gene expression in A549 cells that contribute to EMT following treatment with CSE or TGF-β1, we studied the gene expression profiles of these cells using a human exon array and pathway analysis to identify differentially expressed genes and to predict the upstream regulators involved in the response. This calculation and prediction process compared the profile of differentially expressed gene targets with known profiles stored in database that resulted from the activation or inhibition of upstream regulators. As shown in Table 1, the A549 cells responded to CSE by activating a series of transcriptional factors, including *NUPR1* [42], *TP53* [43,44], *E2F4*, and *E2F6*. CSE treatment likely led to the inhibition of transcriptional regulators such as *FOXM1* [45] and the estrogen receptor. Transcription factors including *BRCA1* [46], *ATF3* [47], *TP63* [48,49], *FOXO1* [45,50], and *HDAC1* [51] were also likely involved in the response to CSE although whether these factors were activated or inhibited was not conclusive. Pathways such as *ERBB2* kinase [52], p38 MAPK, *CDKN1A* [53], *CDK4* [54], lysine-specific demethylase (*KDM5B*) [55], S100 calcium binding protein A6 (*S100A6*) [56], and *TREM1* [57] were also likely activated or inhibited by CSE (Table 1). Most importantly, the activation of the TGF-β1 and TNF pathways was predicted in the response of the A549 cells to CSE treatment. Many of these pathways were previously reported in TGF-β signaling or were reported to be involved in lung cancer (references are placed after gene names).

To validate results of the pathway analysis and to identify the novel molecular pathways that are associated with the CSE-triggered response, the gene expression profile in response to TGF-β1 was compared to the CSE-induced response. As shown in Table 2, the activation of the TGF-β1 and TNF pathways were the first two pathways that were identified by upstream regulator prediction using the gene expression profile of the A549 cells treated by TGF-β1, which was the result of validating the exon array data and pathway analysis. Many previously identified pathways that were determined to be either activated or inhibited in CSE treatment were also identified in the response to TGF-β1, including the estrogen receptor, *ERBB2*, *TREM1*, AR [58], and *TP63*. Genes that were uniquely identified as being potentially activated or suppressed by TGF-β1 treatment included the *JUN*, *WISP2* [59], *IL1A* [60], *IL1B* [60], *HNRNPA2B1* [61], *HDAC6* [62], *SP1* [63], *ITGB1* [64], *CTNNB1* [65], *NEUROG1* [66], *KIAA1524* [67], *TGM2* [68], and *HIF1A* [24,69] pathways. The pathways that are involved in the response to CSE but not in the response to TGF-β1 are discussed below.

### 2.5. ADSC-CM Inhibits Both CSE and TGF-β1 Induced Epithelial Cell Migration

**Figure 6:**
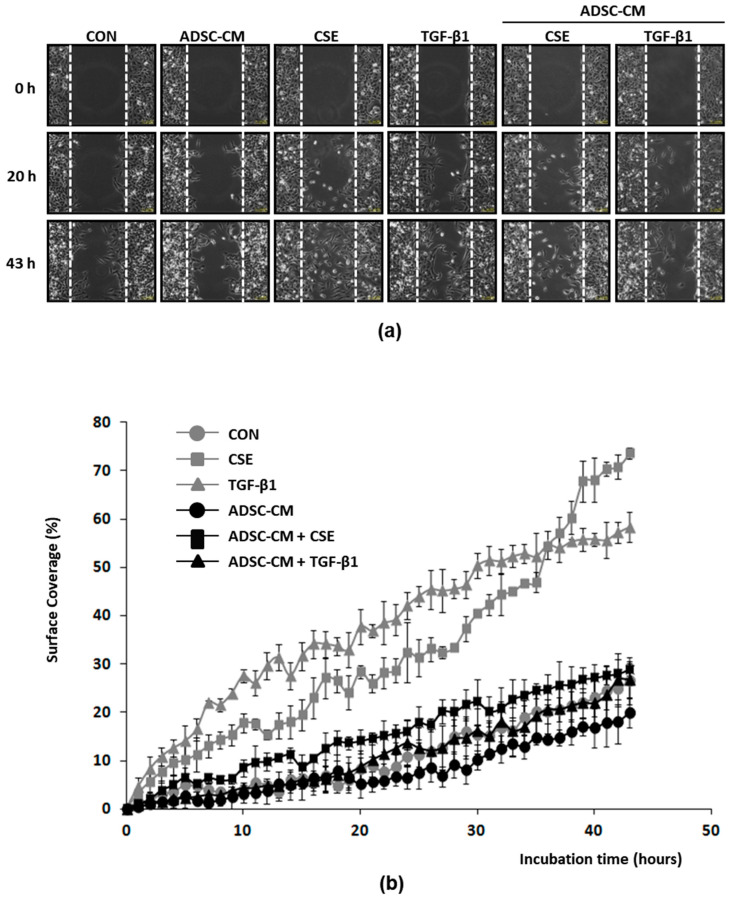
A549 cell migration is increased by CSE and TGF-β1 and is inhibited by ADSC-CM. (**a**) A549 cells migrating into a gap that was 470 μm wide under control, CSE, or 5 ng/mL TGF-β1 stimulation with or without ADSC-CM were captured by a live cell imaging system. Migration images at start (0 h), after 20 h, and after 43 h are shown. Time-lapse images of cell migration are shown in Supplementary data. (**b**) Plot of percentage of surface coverage versus incubation time of A549 cells shows that migration speed was increased by CSE and TGF-β1 and reduced to near control levels by ADSC-CM. *N* = 3. The cell migration speed was further analyzed, and the results are presented in Supplementary Table S1. ADSC, adipose-derived stem cell; CM, conditioned medium; CON, control; CSE, cigarette smoke extract.

ADSC-CM significantly reduced E-cad loss after CSE exposure in both A549 cells and B2B cells and was less effective in reducing the loss of E-cad due to TGF-β1. Higher migratory activities distinguish mesenchymal cells from less mobile epithelial cells; an increase in cell migration also provides strong evidence of epithelial cells acquiring EMT. Therefore, we investigated cell migration after CSE or 5 ng/mL TGF-β1 treatment with or without ADSC-CM. A549 cells were cultured in cell migration assay devices in which cells migrate into a 470 mm wide cell-free gap from both sides, and the process was captured using a live cell imaging system. The rate of cells migrating and covering the cell-free gap was used as an indicator of the speed of cell migration. After 43 h, the A549 control cells covered only about 20% of the gap area, while the CSE- or TGF-β1-treated cells covered 70% or 55% of the gap area, respectively, indicating increasing rates of cell migration by CSE and TGF-β1 (Figure 6a,b). Compared to the A549 cells that had been cultured in α-MEM, the surface coverage after CSE- or TGF-β1-treatment by cell cultured in ADSC-CM was significantly reduced, suggesting an inhibition of CSE- or TGF-β1-induced migration by ADSC-CM. Time-lapse images of the cell migration are shown in videos available as Supplementary data. From the plot of surface coverage versus time (Figure 6b), we identified the most linear phase and the phases that preceded or followed the linear phase, and we observed different cell migration pattern with CSE treatment versus TGF-β1 (Table S1). Unstimulated A549 cells migrated at a speed of 0.29 (% area/h) in the first 18 h (R^2^ = 0.606) and then entered the linear phase, with the speed increasing to 0.81 (%/h) from 18–43 h (R^2^ = 0.979). TGF-β1 resulted in an early increase in the migrating speed to 2.7 (%/h) in the first 7 h (R^2^ = 0.954), and then the speed reduced to 1.1 (%/h) from 7–36 h (linear phase, R^2^ = 0.978), and it reduced further to 0.58 between 36–43 h (R^2^ = 0.834). CSE treatment enhanced cell migration at a speed of 1.2 (%/h) for the first 35 h (R^2^ = 0.981); the speed then increased further to 2.9 (%/h) from 35–43 h (R^2^ = 0.922). Even when stimulated by CSE or TGF-β1, the cells that had been cultured in ADSC-CM displayed a similar migrating speed as the controls. TGF-β1 induced a fast increase in the A549 cell migration speed, which reached a plateau and reduced further after 36 h. The CSE-induced migration was initially slower than TGF-β1, and the migration speed continued to increase. In summary, the results of the analysis support that ADSC-CM significantly inhibits CSE- or TGF-β1-induced EMT and also reduces cell migration. CSE or TGF-β1 treatment resulted in different A549 cell migration patterns, responses that were likely due to activating different sets of molecular pathways. 

## 3. Discussion

Stem cell therapy has been proposed and investigated as a treatment option for COPD, yet the efficacy of stem cell therapy for lung disease has only been demonstrated in preclinical models. Completed clinical trials using MSCs in the treatment of COPD have so far demonstrated adequate safety, but improvements in the treatment outcomes were not conclusive [35]. The results of past trials suggest that the stem cell source, cell number, and route of administration may not be optimized yet and that experimental models of COPD or emphysema do not adequately replicate the context of human disease with complex origins, which is often compounded further by chronic cigarette smoking and exposure to environmental toxins. The mechanisms underlying the pathological progression of COPD are not fully understood, which also contributes to current challenges in finding an effective treatment. In this report, we used CSE collected from sidestream smoke to challenge lung epithelial cells, an experiment that is more relevant to environmental and second-hand smoke exposure that is known to increase risks of COPD or lung cancers. While CSE from mainstream smoke has been frequently used in in vitro studies for establishing the biological consequences of direct smoking and the health risks that smokers experience, very few studies have reported on the biological responses to sidestream smoke [31], which has been reported to be more toxic than mainstream smoke [30].

EMT, a well-characterized process of cellular transformation in cancer development and metastasis, has gained recognition for its extensive implications in chronic airway dysfunction, COPD, pulmonary fibrosis, and lung cancer [70]. As a result of EMT, the highly polarized and tightly packed epithelial cells of the bronchial airway and alveoli become more mesenchymal-like, showing the expression of mesenchymal markers such as vimentin [71]. TGF-β family members, including BMPs, GDFs, endogenous inhibitor FZD receptors, and down-stream mediators such as SMADs, SNAILs, and TWIST, have been shown to be involved in the EMT that occurs in the bronchial epithelial cells of COPD patients or smokers [16,23,26,72,73,74]. It should be noted that most studies showing enhanced EMT have been performed by exposing cells to CSE from mainstream cigarette smoke; EMT-like response in epithelial cells after exposure to sidestream CSE has rarely been reported in previous studies [31], and the details of EMT caused by sidestream CSE have remained mostly unknown. Thus, we need to be careful when directly comparing our results showcasing the upstream regulators that are involved in sidestream CSE with previous studies using mainstream CSE. Their chemical constituents are very different; sidestream CSE often contains heavy metals and the responses that occur after exposure to sidestream cigarette smoke can be more complex than those that result from exposure to mainstream smoke. 

In our study, CSE exposure resulted in cell injury and death in dose- and exposure duration-dependent manners, effects that were inhibited by ADSC-CM. Neither A549-CM nor the ADSC-PT fraction exerted protective effects against CSE exposure, indicating that the beneficial effects that were associated with ADSC-CM were mediated through biomolecules that were larger than 3 kDa in molecular weight and that were secreted by adipose stem cells. Indeed, multiple growth factors have been previously identified from ADSC-CM and MSC-CM [37,38,39]. The depletion of specific factors, such as HGF, VEGF, or SCF, which resulted from the conditioned medium that partially attenuated the protective effects, indicates that multiple growth factors contribute to but that no single component was accountable for most of the ADSC-CM benefits [37,38,39]. We did not further identify any active biomolecules in ADSC-CM that could convey protection and EMT inhibition in this study; however, a weak mitogenic effect that was likely caused by growth factors in ADSC-CM was seen on the A549 cells in culture; this was demonstrated by the fact that ADSC-CM enhanced roughly A549 proliferation by about 30% after 48 h compared to serum free α-MEM, (Figure 2). We showed that cellular responses to CSE likely involve the activation of the TGF-β1 pathway and that ADSC-CM significantly reverses the A549 cell response to CSE and TGF-β1 treatment. TGF-β1 was previously identified as a growth factor in ADSC-CM; thus TGF-β1 may have multiple and complex roles in cell protection and in EMT. In this report, E-cad reduction and vimentin induction in A549 cells or B2B cells responding to CSE, TGF-β1, or ADSC-CM were slightly different. Although different assay methods such as Western blot, immunostaining, or migration assays generated results that were not completely agreeable to each other in great detail, the conclusion that ADSC-CM is capable of reducing EMT induction by CSE or TGF-β1 was consistent. Enhanced EMT by ADSC-CM was reported in glioblastoma [75,76] and lung cancers [77]; the tumor promoting effect caused by ADSCs involves changes in the secretome profile of mesenchymal cells after direct contact with tumor cells [77]. In our results, ADSC-CM alone did not lead to a decrease of E-cad or an increase of vimentin in the A549 or B2B cells (Figure 4b); thus, we do not have any evidence EMT promoting A549 cells by ADSC-CM per se, but we also did not test ADSC-CM in a coculture system containing ADSCs and epithelial cells. Different outcomes may arise from various cell injury models and from studying the EMT as an intrinsic property of epithelial cells or from studying the EMT when it is induced by toxic substances such as CSE. Nevertheless, potential tumor-promoting effects by ADSCs or ADSC-CM need to be carefully addressed in future in vivo studies. 

The fact that the first two pathways that were identified in the cells treated by TGF-β1 by the URA-IPA software were the TGF-β1 and TNF cytokine pathways was an indirect validation of the cell culture, treatment, and array assays as well as the software algorithm and database accuracy. Although an overlap *p* value < 0.01 is considered to be statistically significant according to the algorithm used in the upstream regulator analysis, we only reported high-confidence results with *p* values < 10^−10^. Many of the regulators identified in our study had previously been identified to be associated with TGF-β signaling, EMT, or lung cancer [43,44,45,46,47,48,49,50,51,52,53,54,56,57,58,59,61,62,63,64,65,66,67,68,69]. It should be noted that other regulators not reported here with *p* values between 0.01 and 10^−10^ also have the potential to mediate important cellular responses. Since the roles of TGF-β1 in EMT and the development of COPD and lung cancers have already been discussed previously, we focused on pathways that were activated or inhibited by CSE but that were unchanged by TGF-β1. The roles of TGF-β1-independent regulators in CSE treatment may lead to the distinct cell migration patterns caused by CSE versus TGF-β1. Among those genes listed in Table 1 but not in Table 2, *NUPR1, TP53, CDKN1A, FOXO1, ESR1,* and *HDAC1* were also identified in the response to TGF-β,1 with *p*-values ranging between 0.01 and 10^−10^; thus, these genes should be considered as common regulators. The true unique regulators that were involved in the CSE but not the TGF-β1 responses were *E2F, CCND1, CDK4, FOXM1, KDM5B, S100A6, BRCA1,* and *ATF3*. It is interesting to note that *E2F, CCND1, CDK4*, and *RB*, which has a significant value of *p* = 10^−9^, are interacting signaling networks with well-established roles of regulating the cell cycle, proliferation, angiogenesis, and differentiation [78]. The activation of these pathways may explain excessive cell death and growth inhibition by CSE exposure but not by TGF-β1. *FOXM1* is a cell cycle dependent transcription factor with peak expression in the S and G2/M phases. *FOXM1* has been implicated in carcinogenesis, as nicotine induces *FOXM1* activity [45]. The suppression of *FOXM1* signaling by sidestream CSE may underline a reduction of mitotic cells in the S and G2/M phases, as opposed to an effect by nicotine, a major toxic substance in mainstream CSE. A future study using mainstream CSE as the inducer may validate this hypothesis. *KDM5B* expression was shown increased in lung and other tumors [55]. The finding that CSE causes *KDM5B* activation further implicates that this factor is involved in the promotion of tumor initiation, invasion, and metastasis via epigenetic regulation. *S100A6* itself is an established mesenchymal cell-type marker; the inhibition of the *S100A6* pathway in the CSE-treated cells was unexpected. A recent report also showed that *S100A6* was largely down-regulated in tissues from non-small cell lung cancer patients compared to control tissues [79]. *BRCA1* is a well-established tumor suppressor, and the down-regulation of DNA repair proteins, including *BRCA1*, was implicated in COPD and idiopathic pulmonary fibrosis [80]. The *ATF3* pathway is involved in many cellular processes that are related to fibrosis and to the development of cancer. Increased *ATF3* expression is associated with an increased incidence and invasiveness of non-small cell lung cancer [47]. Taken together, the upstream pathway analysis of differentially expressed genes predicted several pathways that are potentially involved in the epithelial response to sidestream CSE and that related to lung carcinogenesis or COPD.

## 4. Materials and Methods 

### 4.1. Chemicals and Culture Medium

All of the chemicals used in this study were purchased from Sigma-Aldrich (St. Louis, MO, USA), and culture medium and reagents were purchased from Thermo Fisher Scientific (Waltham, MA, USA) unless otherwise specified.

### 4.2. Preparation of ADSC-Conditioned Medium

ADSCs were isolated from the fat pads of young mice (8–12 weeks old) using a previously reported protocol and were cultured in maintenance medium consisting of α-MEM supplemented with 10% fetal bovine serum [81]. ADSC-CM was prepared following a previously described method [40]; in short, ADSCs at 80–90% confluence in 15 cm culture dishes were washed three times with PBS and were cultured with 25 mL fresh serum-free α-MEM for 24 h. The conditioned medium was collected and was passed through a 0.2 μm filter to remove cell debris. The filtered medium was then concentrated 5-fold via ultrafiltration using a 3 kDa cut-off cartridge (GE Healthcare, Chicago, IL, USA) to obtain the ADSC-CM. The pass-through (PT) fraction of the conditioned medium was also collected [40]. To maintain consistency in the media preparations, the A549-conditioned medium (A549-CM), Beas-2- conditioned medium (B2B-CM), and α-MEM used in CSE and TGF-β1 stimulation were also concentrated 5-fold by following the same protocol. All of the media were preserved at 4 °C until use. All animal experiments were conducted in accordance with accepted standards of animal care and were approved by the Institutional Animal Care and Use Committee (NHRI-IACUC-102045A, 23 August 2013; -106099A, 31 July 2017) of the National Health Research Institutes (NHRI), Taiwan.

### 4.3. Cell Injury Induced by Cigarette Smoke Extract Exposure

The sidestream cigarette smoke extracts were prepared from Kentucky Reference Cigarettes 3R4F (University of Kentucky, Tobacco and Health Research Institute, Lexington, KY, USA) [82,83] or from cigarettes of a leading Taiwan brand, Long Life [84], as described previously, using a home-made smoking machine that collects smoke according to the modified Cambridge filter method [82]. The smoke condensates were weighed and were dissolved in DMSO. Human lung epithelial cells (A549, ATCC^®^ CCL-185™) were seeded in 96-well plates at a density of 7000 cells/well and were cultured in α-MEM with 10% FBS overnight. The cells were then exposed to different concentrations of CSE (25 μg/mL, 50 μg/mL, 75 μg/mL, or 100 μg/mL) in serum-free medium for up to 48 h. The WST-1 cell proliferation assay (Clontech-Takara Bio, Mountain View, CA, USA) and the lactate dehydrogenase (LDH) assay (Promega) were conducted to evaluate cell viability and cytotoxicity, respectively. Cell viability (%) in the WST-1 assay was expressed as [OD of Experimental Group]/[OD of Control Group] × 100. In the LDH assay, cytotoxicity (%) was expressed as LDH Released/Maximum LDH × 100 = LDH released/[LDH of cell lysis + LDH released] × 100. Similarly, human non-tumor lung/bronchus epithelial cells (Beas-2B, ATCC^®^ CRL-9609^TM^) were cultured and were maintained in LHC-9 medium (Gibco 12690-013). In studies involving ADSC-CM, the cells were washed three times with PBS and were then incubated in conditioned medium with or without 50 μg/mL CSE, 1–5 ng/mL TGF-β1 (Cell Signaling Technology, Danvers, MA, USA), as in a previously described protocol [85]. Because both the A549 cells and ADSCs were cultured in α-MEM, no substitution of culture medium was required. Beas-2B cells are normally maintained in LHC-9 medium; thus, in experiments involving a comparison of ADSC-CM, α-MEM or Beas-2B-CM require medium substitution using a 50%:50% mixture of LHC-9 medium and α-MEM as the common ground, and only 50% of the volume (the α-MEM portion) was replaced by ADSC-CM. The experiments were performed at least three times.

### 4.4. Gene Expression Analysis

Gene expression analysis using microarray hybridization was performed by the NHRI Microarray Core Lab using Human Exon 1.0 ST Arrays (Affymetrix, Santa Clara, CA, USA). Total RNA taken from cells at 72 h after initiating treatment was isolated using the Qiagen RNAeasy kit, and adequate RNA purity was determined via the absorbance ratios at 260 nm and 280 nm (260/280). Samples with results between 1.85 and 2.01 were processed through quality control and hybridization. The Human Exon 1.0 ST Array contains more than 1.4 million probesets, and at least four probes were targeted to each potential exon sequence in each transcript. The expression of a specific gene transcript was thus redundantly detected so that the expression level was calculated and averaged. Three arrays for each condition using the RNA from three independent studies were used to acquire results for the differentially expressed genes. Ingenuity upstream regulator analysis software (URA; IPA Ingenuity Systems) was used to identify the upstream transcriptional regulators that were involved in the profiles of the differentially expressed genes between the experimental and control groups. The analysis was based on prior gene regulation knowledge stored in the Ingenuity Knowledge Base and was conducted by calculating an overlap *p*-value using Fisher’s Exact test to measure whether there is statistically significant overlap between the identified dataset genes with regulatory potential and the known targets of regulation. The results of the analysis include predictions of upstream regulators, the regulation direction (i.e., activation or inhibition), a *Z*-score assessing the match of the observed and predicted up/down regulation patterns, and a *p*-value measuring the level of significance of overlap between the known targets and the regulatory genes that were identified [86].

### 4.5. Immunoblotting and Fluorescence Detection

Protein extraction and immunoblotting were performed following standard protocols. The following reagents were used: RIPA buffer, proteinase inhibitor (Upstate, Milton Keynes, UK), Bradford reagent (Bio-Rad, Hercules, CA, USA), and antibodies for detecting E-cadherin (Cell Signalling), Vimentin (GeneTex, Irvine, CA, USA), and β-Actin (Merck Millipore, Burlington, MA, USA). For fluorescent immunocytochemistry, A549 or Beas-2B cells were seeded on chamber slides (Nunc Lab-Tek II, Thermo Fisher) for 48 h and, after reaching 70% confluence, were washed three times with PBS, and the cells were then subjected to specified treatments for 72 h. Treatment was followed by methanol fixation and immunostaining with E-cadherin (Santa Cruz) antibody. The following secondary antibodies were used: Alexa Fluor^®^ 488- or Alexa Fluor^®^ 568-conjugated Donkey Anti-Rabbit IgG (H+L; Thermo Fisher). DAPI was used to stain the cell nuclei; cells were viewed, and images were captured using an Olympus BX61 fluorescent microscope. The experiments were performed at least three times.

### 4.6. Cell Migration Assay

A549 cells were seeded in 6-well plates with culture inserts (Ibidi) to create a 470 μm-wide acellular gap between two regions of confluent cells. After removing the culture-insert, cells were washed once with PBS followed by treatment using control or conditioned medium with or without CSE or TGF-β1. Live cell imaging was achieved using a Leica AF6000LX imaging system, which captured one image every 30 min for 43 h. The migration speed of the cells was analyzed by measuring the increase in the area occupied by cells that were migrating into the center gap from both sides using MetaMorph (Molecular Devices, San Jose, CA, USA) and Excel (Microsoft Office). The full area free of cells or the no-cell area at time 0 was set as the baseline, and the complete cell coverage of the same area represented 100%. Percent surface coverage versus time was plotted for each culture condition. The migration study was performed three times.

### 4.7. Statistical Analysis

The data are presented as mean ± SEM, with differences between groups analyzed by Student’s two-tailed *t*-test. *p* < 0.05 was considered statistically significant.

## 5. Conclusions

In this study, we demonstrated that CSE prepared from sidestream cigarette smoke results in cell death and induces EMT in human lung epithelial cells. The protection against cell death and CSE- or TGF-β1-induced EMT conferred by constituents of conditioned medium from stem cell culture was clearly demonstrated. The TGF-β pathway has been targeted in COPD treatment, and we identified several upstream regulators that are involved in the cell response to sidestream CSE exposure that were not activated in response to TGF-β1. These newly identified signaling pathways show potential as novel targets for the development of COPD or lung cancer therapies. Our results add new insight regarding cell injury and EMT induction by toxic substances contained in sidestream CSE and second-hand smoke.

## Figures and Tables

**Table 1:** Predicted upstream regulators in A549 cells treated with cigarette smoke extract.

Upstream Regulator	Molecule Type	Predicted State	z-Score	*p*-Value of Overlap
*NUPR1*	transcription regulator	Activated	9.495	5.38 × 10^−50^
*TP53*	transcription regulator	Activated	6.814	3.51 × 10^−46^
*E2F4*	transcription regulator			1.65 × 10^−38^
*ERBB2*	kinase	Activated	2.186	3.28 × 10^−35^
*TGFB1*	growth factor	Activated	3.746	3.18 × 10^−32^
*FOXO1*	transcription regulator		−1.594	4.04 × 10^−23^
*FOXM1*	transcription regulator	Inhibited	−4.121	5.29 × 10^−21^
E2f	group	Inhibited	−3.317	2.44 × 10^−20^
*CCND1*	transcription regulator		−0.468	7.58 × 10^−20^
*E2F1*	transcription regulator			2.30 × 10^−19^
*CDKN1A*	kinase	Activated	4.263	5.50 × 10^−17^
*CDK4*	kinase			1.78 × 10^−16^
ESR1	ligand-dependent nuclear receptor		−0.519	4.03 × 10^−15^
*TP63*	transcription regulator		−0.619	7.11 × 10^−15^
*KDM5B*	transcription regulator	Activated	4.874	1.41 × 10^−14^
*S100A6*	transporter	Inhibited	−3.742	1.60 × 10^−14^
*TREM1*	transmembrane receptor		0.769	2.85 × 10^−14^
*TNF*	cytokine	Activated	3.127	1.80 × 10^−12^
*E2F6*	transcription regulator	Activated	3.162	7.50 × 10^−12^
P38 MAPK	group		1.399	9.32 × 10^−12^
AR	ligand-dependent nuclear receptor		−1.381	1.51 × 10^−11^
*BRCA1*	transcription regulator		1.564	5.50 × 10^−11^
*HDAC1*	transcription regulator			1.35 × 10^−10^
*ATF3*	transcription regulator		1.134	1.52 × 10^−10^
estrogen receptor	group	Inhibited	−3.329	8.48 × 10^−10^

*TGFB1* (transforming growth factor β-1) and TNF (tumor necrosis factor) pathways are highlighted. *NUPR1*: nuclear protein transcriptional regulator 1, *TP53*: tumor protein p53, *E2F4*: E2F transcription factor 4, E2f: group of mixed types of molecules relating to E2F pathway, ERBB2: v-erb-b2 avian erythroblastic leukemia viral oncogene homolog 2, *FOXO1*: forkhead box O1, *FOXM1*: forkhead box M1, *CCND1*: cyclin D1, *E2F1*: E2F transcription factor 1, *CDKN1A*: cyclin-dependent kinase inhibitor 1A (p21), *CDK4*: cyclin-dependent kinase 4, ESR1: estrogen receptor 1, *TP63*: tumor protein p63, *KDM5B*: lysine (K)-specific demethylase 5B, *S100A6*: S100 calcium binding protein A6, *TREM1*: triggering receptor expressed on myeloid cells 1, *E2F6*: E2F transcription factor 6, P38 MAPK: p38 mitogen-activated protein kinase, AR: androgen receptor, *BRCA1*: breast cancer 1 early onset, *HDAC1*: histone deacetylase 1, *ATF3*: activating transcription factor 3.

**Table 2:** Predicted upstream regulators in A549 cells treated with TGF-β1.

Upstream Regulator	Molecule Type	Predicted State	z-Score	*p*-Value of Overlap
*TGFB1*	growth factor	Activated	6.637	1.43 × 10^−36^
TNF	cytokine		1.508	6.20 × 10^−33^
estrogen receptor	group	Inhibited	−3.797	3.75 × 10^−29^
*ERBB2*	kinase	Activated	4.162	1.27 × 10^−25^
*JUN*	transcription regulator		0.595	7.24 × 10^−25^
*WISP2*	growth factor	Inhibited	−2.947	7.22 × 10^−18^
*TREM1*	transmembrane receptor		−0.563	1.10 × 10^−17^
*IL1A*	cytokine	Activated	2.028	4.32 × 10^−16^
*HNRNPA2B1*	other			6.99 × 10^−16^
ERK	group		1.713	2.47 × 10^−15^
*HDAC6*	transcription regulator	Activated	3.375	3.41 × 10^−15^
*Cg*	complex	Activated	2.661	2.01 × 10^−14^
*SPDEF*	transcription regulator	Inhibited	−3.174	5.92 × 10^−14^
*CTNNB1*	transcription regulator		1.071	6.00 × 10^−14^
P38 MAPK	group		1.489	1.06 × 10^−13^
*COL18A1*	other	Inhibited	−3.022	1.08 × 10^−13^
*SP1*	transcription regulator		0.862	1.57 × 10^−13^
*ITGB1*	transmembrane receptor		0.639	4.14 × 10^−13^
*NEUROG1*	transcription regulator		−1.342	1.42 × 10^−12^
AR	ligand-dependent nuclear receptor		−0.101	2.34 × 10^−12^
*TP63*	transcription regulator		0.650	7.08 × 10^−12^
*IL1B*	cytokine		0.154	9.64 × 10^−12^
*KIAA1524*	other	Inhibited	−2.233	1.04 × 10^−11^
*TGM2*	enzyme		−0.168	1.47 × 10^−11^
*HIF1A*	transcription regulator		1.337	2.12 × 10^−11^

*TGFB1*: TGF-β1, TNF: tumor necrosis factor, *ERBB2*: v-erb-b2 avian erythroblastic leukemia viral oncogene homolog 2, *JUN*: jun proto-oncogene, *WISP2*: *WNT1* inducible signaling pathway protein 2, *TREM1*: triggering receptor expressed on myeloid cells 1, *IL1A*: interleukin 1α, *HNRNPA2B1*: heterogeneous nuclear ribonucleoprotein A2/B1, ERK: mitogen-activated protein kinase, *HDAC6*: histone deacetylase 6, *Cg*: chorionic gonadotrophin, *SPDEF*: SAM pointed domain containing ETS transcription factor, *CTNNB1*: catenin (cadherin-associated protein) β1, P38 MAPK: p38 mitogen-activated protein kinases, *COL18A1*: collagen type XVIIIα1, *SP1*: Sp1 transcription factor, *ITGB1*:integrin β1, *NEUROG1*: neurogenin 1, AR: androgen receptor, *TP63*: tumor protein p63, *IL1B*: interleukin 1β, *TGM2*: transglutaminase 2, *HIF1A*: hypoxia inducible factor 1α.

## Data Availability

No new data were created or analyzed in this study. Data sharing is not applicable to this article.

## Supplementary Materials

The following are available online at https://www.mdpi.com/article/10.3390/ijms222112069/s1.

## Abbreviations

TGF	Transforming Growth Factor
TNF	Tumor Necrosis Factor
ADSC-CM	Adipose Derived Stem Cell-Conditioned Medium
CSE	Cigarette Smoke Extract
COPD	Chronic Obstructive Pulmonary Disease
URA	Upstream Regulator Analysis
EMT	Epithelial-to-Mesenchymal Transition
LDH	Lactate Dehydrogenase
MSC	Mesenchymal Stem Cell
