# Supplementary material for: Conditioned Media of Adipose-Derived Stem Cells Suppresses Sidestream Cigarette Smoke Extract Induced Cell Death and Epithelial-Mesenchymal Transition in Lung Epithelial Cells
Source: Int J Mol Sci. 2021 Nov 8;22(21):12069. doi: 10.3390/ijms222112069 (PMC8584490; doi:10.3390/ijms222112069)
Supplement: Supplementary file 1 [file ijms-22-12069-s001.zip › ijms-1445891-SI/20211017 supplement/Supplement table and file description.pdf]

Supplementary data

Video S1. Time-lapse images of A549 cells cultured in α-MEM migrating into a 470 μm gap

Video S2. Time-lapse images of A549 cells cultured in α-MEM containing 50 μg/ml CSE migrating into a 470 gap

Video S3. Time-lapse images of A549 cells cultured in α-MEM containing 5 ng/ml TGF-β1 migrating into a 470 μm gap

Video S4. Time-lapse images of A549 cells cultured in ADSC-CM migrating into a 470 μm gap

Video S5. Time-lapse images of A549 cells cultured in ADSC-CM containing 50 μg/ml CSE migrating into a 470 μm gap

Video S6. Time-lapse images of A549 cells cultured in ADSC-CM containing 5 ng/ml TGF-β1 migrating into a 470 μm gap

Table S1

		**Control**	**CSE**	**TGF-β1**
**Pre-linear Phase**	Hour	0-18	--	0-7
	Speed (% area/h)	0.293	--	2.7
	R^2	0.606	--	0.954
**Linear Phase**	Hour	18-43	0-35	7-36
	Speed (% area/h)	0.811	1.198	1.143
	R^2	0.979	0.981	0.978
**Second Linear Phase**	Hour	--	35-43	36-43
	Speed (% area/h)	--	2.936	0.58
	R^2	--	0.922	0.834
